# Clinical, Laboratory and Imaging Characteristics of Hospitalized COVID-19 Patients with Neurologic Involvement; a Cross-Sectional Study

**DOI:** 10.22037/aaem.v10i1.1507

**Published:** 2022-01-30

**Authors:** Ali Zare Dehnavi, Mohammadreza Salehi, Mehran Arab Ahmadi, Mohammad Hossein Asgardoon, Farzad Ashrafi, Nasrin Ahmadinejad, Atefeh Behkar, Ramin Hamidi Farahani, Hassan Hashemi, Abbas Tafakhori, Hamze Shahali, Mohammad Rahmani, Alireza Ranjbar Naeini

**Affiliations:** 1 Department of Neurology, School of Medicine, AJA University of Medical Sciences, Tehran, Iran.; 2 Infectious Diseases and Tropical Medicines Department, Tehran University of Medical Sciences, Tehran, Iran.; 3 Advanced Diagnostic and Interventional Radiology Research Center, Tehran University of Medical Sciences, Tehran, Iran.; 4 School of Medicine, Tehran University of Medical Sciences, Tehran, Iran.; 5 Functional Neurosurgery Research Center, Shohadaye Tajrish Neurosurgical Center of Excellence, Shahid Beheshti University of Medical Sciences, Tehran, Iran.; 6 Department of Infectious Disease, AJA University of Medical Sciences, Tehran, Iran.; 7 Department of Neurology, School of Medicine, Imam Khomeini Hospital, Tehran University of Medical Sciences, Tehran, Iran.; 8 Department of Aerospace and Sub Aquatic Medicine, AJA University of Medical Sciences, Tehran, Iran.

**Keywords:** COVID-19, Neurology, Neurologic Manifestations, Neuroimaging, Tomography, X-ray computed, Magnetic Resonance Imaging, Risk Factors

## Abstract

**Introduction:**

Although neurologic involvement and neuroimaging abnormalities have been frequently identified in COVID-19 patients, the underlying factors remain unclear. In this study, we assessed the association of the neurological manifestations and neuroimaging features of hospitalized COVID-19 patients with their clinical, laboratory, and imaging characteristics.

**Methods:**

This multicenter cross-sectional study was conducted between September 2020 and March 2021 at two large academic hospitals in Tehran, Iran. We used census sampling from medical records to enroll hospitalized patients with a positive COVID-19 Polymerase chain reaction (PCR) test who underwent brain imaging due to presenting any acute neurologic symptom during hospital stay.

**Results:**

Of the 4372 hospitalized patients with COVID-19, only 211 met the inclusion criteria (35.5% with severe infection). Central nervous system and psychiatric manifestations were significantly more common in severe cases (p ≤ 0.044). Approximately, 30% had a new abnormality on their neuroimaging, with ischemic (38/63) and hemorrhagic (16/63) insults being the most common. The most frequent reasons that provoked cranial imaging were headache (27%), altered consciousness (25.6%), focal neurologic signs (19.9%), and delirium (18%). Analysis revealed a positive correlation for age, neutrophilia, lymphopenia, erythrocyte sedimentation rate (ESR), and C-reactive protein (CRP) with the emergence of neuroimaging abnormalities (p ≤ 0.018). In addition, patients with new neuroimaging abnormalities had a significantly higher lung CT score than those without any pathologic findings (11.1 ± 4.8 vs. 5.9 ± 4.8, p < 0.001).

**Conclusion:**

Approximately 30% of the study population had various acute neuroimaging findings. The lung CT score, neutrophil count, and age were strong predictors of acute neuroimaging abnormalities in hospitalized COVID-19 patients.

## 1. Introduction:

COVID-19 has been declared a public health emergency of international concern by the World Health Organization (WHO) (1). According to WHO statistics, to date (December 20, 2021), this disease has infected roughly 272 million people worldwide and has led to more than 5 million deaths (2). According to WHO statistics, Iran is one of the top 20 nations with the highest prevalence of COVID-19, with approximately 6.2 million individuals infected and 130,000 deaths documented so far (December 20, 2021) (2). COVID-19 can have a variety of clinical manifestations, from asymptomatic to death (3). With the increase in COVID-19 cases globally, many extra-pulmonary manifestations of this disease, such as neurologic ones, have been documented (3, 4). Different investigations have found that the prevalence of at least one new-onset neurological manifestation linked to COVID-19 infection is highly variable, ranging from around 10% to more than 80% (5-7). In addition, neuropsychiatric problems such as delirium have been frequently documented in hospitalized patients, and linked to a higher mortality rate in COVID-19 cases (8, 9). Several studies have also documented neuroimaging abnormalities in patients with COVID-19, including ischemic and hemorrhagic infarction, cerebral venous thrombosis, demyelinating disorders such as acute disseminated encephalomyelitis (ADEM), meningitis, encephalomyelitis, acute hemorrhagic necrotizing encephalopathy (ANE), and hemorrhagic posterior reversible encephalopathy syndrome (PRES) (4, 10-12). 

Due to the neurological symptoms that emerge throughout the disease period, the potential effects of SARS-COV-2 on the nervous system has attracted remarkable attention, and several possible mechanisms of neurological injury have been postulated (13). This virus can affect the central nervous system via olfactory nerves or, enter brain cells by binding to angiotensin-converting enzyme-2 (ACE-2) or cause neuroinflammation following a cytokine storm (3, 14). COVID-19 can affect the central nervous system, peripheral nervous system, and musculoskeletal system, leading to various neurological manifestations including headache, anosmia, ageusia, dizziness, altered consciousness, myalgia, myelopathy, encephalopathy, meningitis, seizure, syncope, hemorrhage, and stroke (15, 16). 

Despite the rapidly growing literature on this subject, correlations between neurological symptoms and/or neuroimaging findings in COVID-19 and other variables are still mostly unknown. Only a few studies have investigated the association between neurological symptoms and other variables. Our objective in this study is to evaluate COVID-19-related neurological and neuroimaging findings in hospitalized patients, while investigating their relationship with various clinical, laboratory, and lung CT score characteristics. 

## 2. Methods:

***2.1 Study design and setting***

This cross-sectional study was conducted between September 22, 2020 and March 30, 2021 at Imam Khomeini Hospital Complex and Shohadaye Tajrish Medical Center in Tehran, Iran. We used the STROBE checklist as the reporting guideline for this study. This was a retrospective study and all admission, discharge, diagnostic, and therapeutic decisions were made based on the latest version of the national COVID-19 protocol during the study, and we did not interfere with the patient's diagnostic process and didn't charge the patient or the system anything. The study protocol was approved by the ethics committee of AJA University of Medical Sciences, receiving the ethics code number (IR.AJAUMS.REC.1399.163). 

***2.2 Study population***

- All adult (≥18 years old) hospitalized COVID-19 patients with positive real-time reverse transcription-polymerase chain reaction (RT -PCR) test and a neuroimaging study (including brain and/or spine imaging) following the emergence of any acute neurologic manifestation during hospital stay were included in the study. Exclusion criteria were: (a) known history of previous neurological disorders; (b) previous neuroimaging abnormalities; (c) neurologic manifestation with non-COVID-19 etiology; and (d) incomplete medical records, which failed to meet the requirements for our checklist.

Acute neurologic manifestations included: headache, dizziness, altered consciousness, seizure, any focal neurologic symptoms, delirium, psychosis, and any other type of neuropathy.

***2.3. Outcomes and measurements***

A checklist was designed and developed to extract patients’ data. The collected data included demographic characteristics (age, gender, and underlying disease), clinical features (degree of severity on admission (measured using American Thoracic Society guidelines for community-acquired pneumonia (17), severe or non-severe), outcome (death or discharge), neurologic/psychiatric manifestations, and indication for neuroimaging), initial laboratory data (included a complete blood cell count (CBC), and assessment of renal function, C - reactive protein (CRP), erythrocyte sedimentation rate (ESR), creatine kinase (CK), and lactate dehydrogenase (LDH) according to the clinical care needs of the patient), chest CT scan, and neuroimaging findings.

All neurological symptoms in this study were evaluated by an expert neurologist after suspicion of a clinician during daily routine practice in the mentioned centres. Neurologic manifestations were divided into three groups: central nervous system (CNS) manifestations (dizziness, headache, impaired consciousness, acute cerebrovascular disease, ataxia, and seizure), peripheral nervous system (PNS) manifestations (taste impairment, smell impairment, vision impairment, and nerve pain), and acute psychiatric manifestations (psychosis and delirium). Both neurological and psychiatric symptoms were extracted from the consultation notes of experienced neurologists and psychiatrists. Acute cerebrovascular disorders including ischemic or hemorrhagic insults were diagnosed by clinical symptoms and brain imaging. Seizure was diagnosed based on clinical symptoms at the time of presentation.

Indications for neuroimaging were also extracted from medical records and were categorized into six groups: 1) focal neurologic signs (including stroke, transient ischemic attack (TIA) and all possible forms); 2) altered consciousness / reduced GCS; 3) delirium; 4) headache; 5) seizure; and 6) miscellaneous.

***2.4. Image acquisition and interpretation***

We obtained all the images in our study as per standard of care protocols. 1.5-T scanners (Siemens Avanto, Germany) with standardized protocols were utilized for brain and spine magnetic resonance imaging (MRI) scans. All CT and MRI images were initially reviewed by two experienced neuroradiologists (each having at least ten years of neuroradiology experience), and any disagreements were settled by consensus.

All available chest CT scans were evaluated for CT lung severity score via lobar based assessment (18). Each of the five lung lobes was subjectively graded from 0 to 5 (0, no involvement; 1, involvement < 5%; 2, involvement 6–25%; 3, involvement 26–50%; 4, involvement 51–75%; 5, involvement > 75%) in lobar based evaluation. The total score was the sum of the individual lobar scores and ranged from 0 to 25. All neuroimaging was analysed for the following characteristics: 1) ischemic insults; 2) haemorrhagic insults; 3) leptomeningeal or cranial nerves enhancement; 4) cerebral venous thrombosis; 5) acute encephalopathy; 6) white matter involvement and any other new abnormal findings.

***2.5 Statistical analysis***

All statistical analyses were conducted using SPSS version 20. Mean and standard deviation were used for reporting normally distributed quantitative variables; Median and interquartile range (IQR) were used for reporting quantitative variables that were not normally distributed, and frequency (percentage) was used to report categorical variables.

Independent sample t-test or Mann Whitney test was used for comparing two quantitative groups based on the result of Shapiro-Wilks for normality. Chi square test, and if needed Fisher’s exact test was used to evaluate the association between two categorical variables. We also performed multivariate binary logistic regression analysis on factors that significantly correlated with neuroimaging abnormality. P-values < 0.05 were considered statistically significant.

## 3. Results:

***3.1 Demographic, clinical and laboratory characteristics***

During the study period, a total of 4372 hospitalized patients with SARS-CoV-2 infection were identified. Of these, 211 patients met our inclusion criteria (52.6% male). Their mean age was 60.7 (standard deviation (SD) =15.8) years (age range, 18-94 years). Based on American Thoracic Society guidelines for community-acquired pneumonia, 75 (35.5%) of cases were categorized as severe COVID-19 infections and 136 (64.5%) of them were non-severe. Patients’ characteristics are presented in Table 1.

 84.4%, 19.4%, and 35.1% of the patients showed at least one CNS, PNS, and neuropsychiatric manifestation, respectively. CNS findings were the most prevalent neurologic symptoms overall, with a significantly higher prevalence in the severe group (93.3% vs. 80.1%, p = 0.011). The most frequently recorded CNS manifestations were: headache (40.3%), reduced consciousness (36%), and focal neurologic symptoms (18%). Altered consciousness, focal neurologic findings, and seizures were more prevalent in severe infections compared to non-severe infections; headache was significantly higher in non-severe infections (29.3% vs. 46.3%, p = 0.016). Neuropsychiatric manifestations were also fairly common, with a total prevalence of around 35%, and were significantly associated with infection severity (severe (44.0%) vs. non-severe (30.1%), p = 0.044). PNS manifestations were the least common among these three categories, with an overall prevalence of about 20% and no remarkable difference between severe and non-severe groups. In the PNS group, 2 cases were also diagnosed with Guillain-Barre syndrome. Clinical manifestations of patients are detailed in Table 1.

In the comparison of various factors between severe and non-severe cases, patients with severe infection were significantly older (64.5±14.2 vs. 58.6±16.4, p = 0.010), and had a higher mortality rate (p <0.001). In addition, past medical history of hypertension (54.7% vs. 39.7%, p = 0.037) also associated with severity. However, no other difference was observed between these two groups. 

Regarding laboratory tests, patients with a severe infection had a higher inflammatory response, including higher neutrophil counts, lower lymphocyte counts, increased C-reactive protein levels, elevated erythrocyte sedimentation rate, and higher lactate dehydrogenase levels (p ≤ 0.010) compared to those with non-severe infection. During the study, 13 patients underwent lumbar puncture and their cerebrospinal fluid findings are shown in Table 1.

***3.2 Neuroimaging findings***

In the study population, 160 (75.8%) were examined using brain CT, 5 (2.4%) underwent brain and/or spine MRI, and 46 (21.8%) underwent both CT and MRI. Apart from changes commonly found in elderly patients, neuroimaging indicated no major abnormalities in 148 (70.1%) participants. Abnormal findings were seen in 63 (29.9%) cases, with the rate of abnormality being significantly higher in patients with severe COVID-19 infection (52 % (severe) vs. 17.6% (non-severe), p < 0.001). 

**Figure1:**
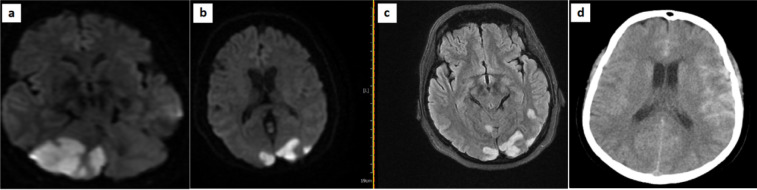
**(a, b, c) **Acute infarct in posterior cerebral artery (PCA) territory with restriction on diffusion-weighted magnetic resonance imaging (DWI) in a 53-year-old female with COVID-19 on the ninth day of admission; **(d)** hyper dense materials in brain sulci more prominent on left peritoneal lobe in favor of Subarachnoid hemorrhage (SAH)

The main neurologic imaging hallmark was acute ischemic infarcts, found in 38 (18%) of the 211 individuals. Of these, 35 (92%) had territorial infarction and 3 (8%) had non-territorial infarcts. Ischemia in the territory supplied by the middle cerebral artery (MCA) (27/35, 77%) was the most prevalent among territorial infarcts. The rest included: 3 posterior cerebral artery (PCA), 1 anterior cerebral artery (ACA), 2 infratentorial, 1 PCA+ACA, and 1 PCA +infratentorial (figure 1). 

Intracranial hemorrhages (ICHs) were the second most common finding (16/211), with micro-hemorrhages being the most common (8/16, 50%), followed by 5 large cranial hemorrhages and 3 cases of subarachnoid hemorrhage (SAH) (figure 1). Of these, one was a 27-year-old female with no remarkable past medical history surveyed with complaints of severe headache and altered mental status approximately one week after the beginning of COVID-19 symptoms who underwent brain CT and MRI. Cranial imaging revealed brain edema and a 12×9×8mm mass in the left aspect of the pituitary fossa with a hemorrhagic appearance suggestive of pituitary adenoma apoplexy.

**Figure2:**
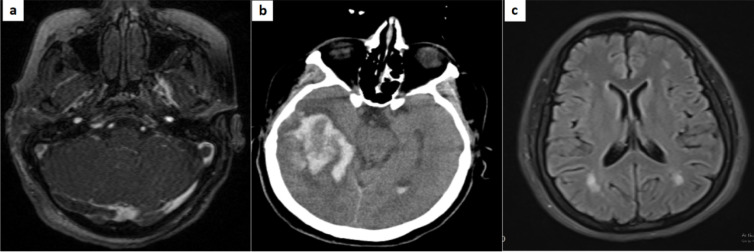
**(a, b) **Large hyper dense heterogeneous lesion in right temporal lobe with peripheral edema, more evaluated with brain magnetic resonance imaging/venography (MRI/MRV), which showed abnormal signal in right sigmoid sinus compatible with cerebral venous thrombosis; **(c)** T2 Flair images in a 39-year-old female with COVID-19 shows some hyper intense predominantly subcortical and deep white matter lesions without periventricular and corpus callosum involvement suggestive of acute disseminated encephalomyelitis (ADEM).

Seven cases were diagnosed with cerebral venous thrombosis (CVT), one of which had superior sagittal sinus thrombosis accompanied by leptomeningeal enhancement. Another case was a 66-year-old man with hypertension, classified as a severe infection, who underwent cranial imaging due to decreased consciousness and seizure. His brain MRI showed an abnormal signal area with hemorrhagic change in the right temporal lobe and an abnormal signal in the right sigmoid sinus favoring venous infarct due to dural venous sinus thrombosis. In MRV (Magnetic Resonance Venography), transverse and sigmoid sinus was not seen, and abnormal signals in T2/W sequences consistent with venous thrombosis were present (figure2). Details of neuroimaging characteristics are summarized in Table 2.

***3.3 Neuroimaging indications***

**Figure3:**
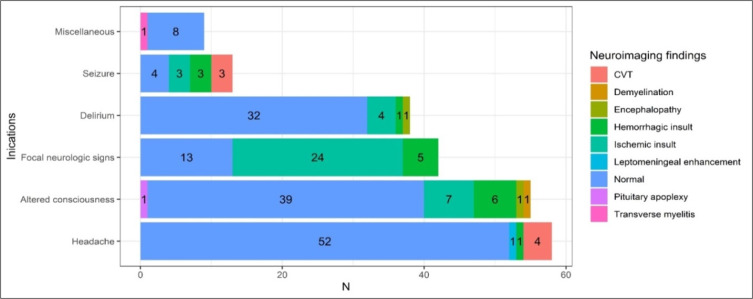
Distribution of neuroimaging findings based on neuroimaging indications. CVT: cerebral venous thrombosis

Among reasons for undergoing imaging, the most common indications were headache (27%), impaired mental status (25.6%), and focal neurologic signs (19.9%) (Table2). Indications for neuroimaging matched with neuroimaging characteristics are presented in figure 3. Most of the patients with headache (52/57, 91%) and delirium (32/38, 84%) had no abnormal findings on neuroimaging, but most of those who had seizures (7/11, 64%) had pathologic findings on neuroimaging. Most ischemic or hemorrhagic insults were seen among patients who underwent neuroimaging due to altered consciousness or focal neurology (42/55, 76%). On the other hand, all CVT cases were detected in people who had headaches and/or seizures.

***3.4 Association of neuroimaging with clinical, laboratory features, and lung CT score***

Table 3 presents the characteristics of patients with and without neuroimaging abnormalities. We found that individuals with abnormal findings in neuroimaging studies were significantly older (p = 0.009) and had a higher level of ESR, CRP, and neutrophil count (p ≤ 0.018). The analysis also revealed that chest CT score of patients with COVID-19 who had new abnormalities on neuroimaging was significantly higher than those who didn’t have any pathologic neuroimaging findings (mean CT score±SD, 11.1±4.8 vs. 5.9±4.8, p < 0.001). However, no other related factor was detected.

***3.5 Predictors of*** ***neuroimaging abnormality***

The multivariate logistic regression on the factors influencing neuroimaging abnormality is presented in Table 4. This analysis showed that age (B=0.041, SE=0.013, Exp(B)=1.042, p = 0.002), neutrophil count (B=0.000, SE=0.000, Exp(B)=1, p = 0.039) and lung CT Score (B=-0.181, SE=0.045, Exp(B)=0.834, p = 0.000) were strong predictors of neuroimaging abnormality. However, ESR, CRP, and lymphocyte count showed no significant prediction ability for neuroimaging abnormality (p ≥ 0.116).

**Table1:** Clinical and laboratory findings of patients based on severity of infection

**Characteristics**		**Total (N= 211)**	**Severity of infection**		**P**
			**Severe n=75**	**Non-severe n=136**	
Age (year)		60.7±15.8	64.5±14.2	58.6±16.4	**0.010**
Neuroimaging abnormality		63(29.9)	39(52)	24(17.6)	**<0.001**
**Gender**	Male	111(52.6)	39 (52)	72(52.9)	0.896
	Female	100 (47.4)	36 (48)	64(47.1)	
**Comorbidities**	Hypertension	95(45)	41(54.7)	54(39.7)	**0.037**
	Diabetes mellitus	81(38.4)	26(34.7)	55(40.4)	0.409
	Heart disease	51(24.2)	17(22.7)	34(25.0)	0.705
	COPD	10(4.7)	4(5.3)	6(4.4)	0.763
	CKD	14(6.6)	8(10.7)	6(4.4)	0.081
	Liver disease	7(3.3)	3(4.0)	4(2.9)	0.681
	Malignancy	18(8.5)	8(10.7)	10(7.4)	0.409
	Tobacco smoking	26(12.3)	9(12.2)	17(12.5)	0.916
**Outcome**	Discharged	158(74.9)	38(50.7)	120(88.2)	**<0.001**
	Expired	53(25.1)	37(49.3)	16(11.8)	
**CNS **	Total	179(84.4)	70(93.3)	109(80.1)	**0.011**
	Dizziness	33(15.7)	15(20)	18(13.2)	0.195
	Headache	85(40.3)	22(29.3)	63(46.3)	**0.016**
	LOC	76(36.0)	46(61.3)	30(22.1)	**<0.001**
	Ataxia	22(10.4)	9(12.0)	13(9.6)	0.579
	Seizure	11(5.2)	9(12.0)	2(1.5)	**0.001**
	Focal neurologic findings	38(18.0)	23(30.7)	15(11.0)	**<0.001**
	Encephalopathy	4(1.9)	3(4.0)	1(0.7)	0.096
**PNS**	Taste impairment	23(10.9)	3(4.0)	20(14.7)	**0.017**
	Smell impairment	24(11.4)	3(4.0)	21(15.4)	**0.012**
	Visual impairment	10(4.7)	5(6.7)	5(3.7)	0.328
	Guillain-Barre syndrome	2(0.9)	1(1.3)	1(0.7)	0.668
**Psychiatric**	Total	74(35.1)	33(44.0)	41(30.1)	**0.044**
**Laboratory **	WBC (cells /μL)	10219±4932	10930±6344	9827±3917	0.174
	Neutrophil (cells /μL)	7904±4353	9271±5569	7150±3295	**0.003**
	Lymphocyte (cells /μL)	1586±963	933±643	1945±921	**<0.001**
	Platelet (cells /μL)	235004±114506	212413±102995	247463±118935	**0.033**
	ESR (mm/hr)	43.0±33.1	62.1±26.9	32.4±31.4	**<0.001**
	C-reactive protein (mg/L)	37.4±29.0	55.4±23.9	28.0±27.3	**<0.001**
	CPK (U/L)	151.1±345.9	192.8±183.7	128.6±406.3	**0.001**
	Lactate dehydrogenase (U/L)	543.2±436.9	661.3±585.4	478.7±313.7	**0.01**
	Blood urea nitrogen (mg/dL)	56.3±49.2	70.8±53.1	48.3±45.2	**0.002**
	Creatinine (mg/dL)	1.5±1.0	1.6±1.1	1.3±0.9	0.075
**CSF **	High WBC (cells/mm3)	4/13	----	----	
	Increased Protein (mg/dL)	5/13	----	----	

Data presented as mean ± standard deviation (SD) or number (%). ESR: Erythrocyte sedimentation rate; LOC: loss of consciousness; CPK: Creatine phosphor Kinase; WBC: White blood cell; CKD: chronic kidney disease; COPD: chronic obstructive pulmonary disease.

**Table2:** Summary of patients’ neuroimaging (computed tomography scan or magnetic resonance imaging) findings

**Variables**	**Values n (%)**
**Indication for neuroimaging**	
Headache	57 (27.0)
Altered consciousness	54 (25.6)
Focal neurologic signs	42 (19.9)
Delirium	38 (18.0)
Seizure	11 (5.2)
Miscellaneous	9 (4.3)
**Ischemic insult**	
Territorial	35 (16.6)
Non-territorial	3 (1.4)
**Hemorrhagic insult**	
Large intracranial and intraventricular	5 (2.4)
Microhemorrhage	8 (3.8)
Subarachnoid hemorrhage (SAH)	3(1.4)
**Territory of ischemic insult**	
Middle cerebellar artery (MCA)	27(12.8)
Posterior cerebellar artery (PCA)	5(2.4)
Anterior cerebellar artery (ACA)	2(0.9)
Infratentorial	3(1.4)
**Other findings**	
Acute encephalopathy	2 (0.9)
Leptomeningeal enhancement	1 (0.5)
Pituitary apoplexy	1(0.5)
Cranial nerves	0 (0.00)
Cerebral venous thrombosis (CVT)	7 (3.3)
Transverse myelitis	1 (0.5)
Demyelination (white matter involvement)	1 (0.5)

**Table 3:** Comparing the patients’ characteristics between cases with and without acute neuroimaging abnormality

**Characteristics**	**Total n=211**	**Neuroimaging abnormality **		**P**
		**With n=63**	**Without n=148**	
**Age (years) **				
Mean ± SD	60.7 ± 15.8	56.3 ± 16.1	62.6 ± 15.4	**0.009**
**Gender**				
Male	111 (52.6)	28 (44.4)	83 (56.1)	0.121
Female	100 (47.4)	35 (55.6)	65 (43.9)	
**Comorbidities**				
Hypertension	95 (45.0)	30 (47.6)	65 (43.9)	0.621
Diabetes	81 (38.4)	22 (34.9)	59 (39.9)	0.499
Heart disease	51 (24.2)	13 (20.6)	38 (25.7)	0.434
COPD	10 (4.7)	1 (1.6)	9 (6.1)	0.160
Chronic kidney disease	14 (6.6)	6 (9.5)	8 (5.4)	0.271
Liver disease	7 (3.3)	0 (0.00)	7 (4.6)	0.079
Malignancy	18 (8.5)	4 (6.3)	14 (9.5)	0.459
Tobacco smoking	26 (12.3)	10 (15.9)	16 (10.8)	0.306
**Outcome**				
Discharged	158 (74.9)	42 (66.7)	116 (78.4)	0.073
Expired	53 (25.1)	21 (33.3)	32 (21.6)	
**Laboratory findings**				
WBC (cells/μL)	10219±4932	11090±5806	9849±4480	0.094
Neutrophil (cells /μL)	7904 ± 4353	9158 ± 5094	7371 ± 3894	**0.006**
Lymphocyte (cells /μL)	1586 ± 963	1238 ± 714	1733 ± 1017	**<0.001**
Platelet (cells /μL)	235004±114506	247349±105979	229750±117901	0.308
ESR (mm/hr)	43.0 ± 33.1	52.7 ± 27.5	39.0 ± 34.3	**<0.001**
CRP (mg/L)	37.4 ± 29.0	44.3±26.3	34.4 ± 29.7	**0.018**
CPK (U/L)	151.1 ± 345.9	214.7±592.2	121.5 ± 107.3	0.235
LDH (U/L)	543.2 ± 436.9	518.1±358.0	554.0 ± 467.5	0.632
BUN (mg/dL)	56.3 ± 49.2	50.0±37.3	59.0 ± 53.4	0.166
Creatinine (mg/dL)	1.5 ± 1.0	1.2 ± 0.6	1.5 ± 1.1	**0.027**
**CT lung severity score (0-25)**				
Mean ± SD	7.4 ± 5.3	11.1 ± 4.8	5.9 ± 4.8	**<0.001**

Data presented as mean ± standard deviation (SD) or number (%). WBC: White blood cell; CT: computed tomography scan; ESR: Erythrocyte sedimentation rate; CRP: C-reactive protein; CPK: Creatine Phosphokinase; LDH: Lactate dehydrogenase; BUN: Blood urea nitrogen; COPD: Chronic obstructive pulmonary disease.

**Table4:** The multivariate binary logistic regression of the potential factors predicting neuroimaging abnormality

**Parameters**	**B**	**Standard error**	**EXP (B)**	**P value**
**Constant**	-0.175	0.954	0.839	0.854
**Age**	0.041	0.013	1.042	**0.002**
**Neutrophil**	0.000	0.000	1.000	**0.039**
**Lymphocyte**	0.000	0.000	1.000	0.116
**Erythrocyte sedimentation rate (ESR)**	-0.006	0.009	0.994	0.486
**C reactive protein (CRP)**	0.004	0.010	1.004	0.685
**Creatinine**	0.369	0.318	1.446	0.246
**CT lung severity score**	-0.181	0.045	0.834	**0.000**

CT: computed tomography.

## 4. Discussion:

In this study, we surveyed hospitalized patients with COVID-19-related neurologic symptoms requiring neuroimaging, focusing on their clinical, laboratory, and chest CT scan characteristics. Our patients had a wide range of neurologic symptoms as well as neuroimaging indications and findings. We discovered that nearly 30% of COVID-19 patients with neurological involvement had an abnormality in their neuroimaging, with the most commonly reported abnormality being acute ischemic infarcts, followed by ICH. Analysis showed that the emergence of acute neuroimaging findings was related to a higher lung CT severity score as well as age, neutrophil count, lymphocyte count, ESR, and CRP level. Multivariate logistic regression on the factors influencing neuroimaging abnormality identified age, neutrophil count, and lung CT score as strong predictors of new abnormal neuroimaging findings. We also found that being old; having a past medical history of HTN, having a CNS manifestation, and having neuropsychiatric symptoms are associated with disease severity. 

Patients with hypertension in our study were more likely to have severe COVID-19 infection; similar to the findings of several previous papers (19-22) which found that hypertension is related to a greater risk of mortality. So, this underlying condition should be considered by clinicians as a predictor of progression of COVID-19 to severe status and poor outcome.

Patients with severe infection were found to be older. According to some studies (19, 21, 22), older patients had a greater mortality rate. Patients with abnormal neuroimaging were also found to be older, which is consistent with the findings of Chen et al., who reported that age is associated with acute cerebrovascular events in COVID-19 patients (20). This is important because in elderly patients with COVID-19, who have nonspecific symptoms of neurological involvement, the likelihood of neurological involvement should always be considered, and the threshold for neurological imaging should be lower. However, in contrast to some investigations that found a higher prevalence of severe cases in men, no gender difference was observed between these two groups in our study (23-25).

CNS manifestations were shown to be the most prevalent neurological manifestation, with headache being the most common (40.3%). This finding is in agreement with previous studies, which found headache to be one of the most common neurological manifestations, with a frequency ranging from 4 to more than 40% (6, 7, 16, 19, 21, 22, 26). We found that almost 30% of COVID-19 patients with neurologic manifestations had abnormal neuroimaging. This number has been observed to range from 20% to more than 80% in different studies (26-32). The disparities could be due to the lower threshold for undergoing brain imaging in Iran’s health system setting, sample-size differences, or differences in the characteristics of the sample groups. For example, in the study that reported this number to be above 80%, a greater percentage of the sample experienced more serious neurological symptoms, such as paresis or loss of consciousness, or had more comorbidities. Like Mahammedi et al.(32), we observed that patients who had acute abnormalities on neuroimaging had a significantly higher CT lung severity score. Although further research is needed to verify this association, it suggests that any neurological symptoms in COVID-19 patients with a high CT lung severity score should be taken seriously. In addition, we can employ the CT lung severity score as a prognostic tool in managing COVID-19 patients with neurological manifestations.

Ischemia and infarction were the most common imaging abnormalities, as they had been in many earlier studies (19, 28, 30, 33, 34). However, we have an inadequate understanding of the mechanisms of the neurologic manifestations presented in COVID-19 patients, and we don’t know whether they were caused by direct invasion of the coronavirus to the central nervous system (35). SARS-CoV-2 has been demonstrated to enhance coagulopathy in previous investigations (36, 37), thus finding ischemia and infarction in neuroimaging appears to be a possibility that should be considered. We also reported four cases of encephalopathy, two of which displayed encephalopathy features on brain imaging. Both were in the severe group and had to undergo neuroimaging due to delirium and focal neurologic signs; tragically, one of them passed away during the hospital stay. We also described a case of pituitary apoplexy in a young woman, which has been recorded in only a few cases in the COVID-19 setting (38). 

There are some limitations to this study that should be highlighted. Even though our sample was large and multi-center, we only investigated hospitalized patients in two large hospitals. Our study was retrospective, which can contribute to an underestimation of variable frequency. Multinational and outpatient studies on long-term outcomes as well as other study designs should be considered. Due to the subjective nature of neuroimaging and chest CT scan findings, it was challenging to standardize them. We overcame this constraint by having two expert neuroradiologists review all CT and MRI images and reaching a consensus on any disputes. Another major limitation was that we only enrolled COVID-19 patients with neurologic manifestations who underwent neuroimaging. Because performing neuroimaging on all patients as a routine is unnecessary and immoral, increasing the probability of exposure to the virus during a pandemic, imaging was done selectively in patients with more serious and significant neurologic symptoms.

## 5. Conclusion:

Our study demonstrates that roughly 30% of the studied cases had various new neuroimaging abnormalities, which should not be dismissed during the COVID-19 pandemic. Furthermore, age, neutrophil count, and lung CT score were shown to be strong predictors for the emergence of neuroimaging pathologic findings. 

## 6. Declarations:

### 6.1 Acknowledgments

We thank all the medical team at the neurology, radiology, infectious disease, and emergency medicine departments of both hospitals, including doctors, nurses, the health care experts, and the staff. We also thank the patients and their families for their cooperation.

### 6.2 Funding and support 

None 

### 6.3 Conflict of interest

The authors declare no conflict of interest

### 6.4 Data Availability

The datasets generated and analyzed during the current study are available from the corresponding author on reasonable request.

### 6.5 Authors’ contribution

Design of the study by AZ, MS, A.R and F.A; Data acquisition by AZ, MA, MR and A.T; Images review by HH, NA, and MA, Data analysis and interpretation by MHA; AR, FA, and RH; drafting the manuscript by AZ, MHA, HS, and AB; Revision of the manuscript by MS, AR, FA, MA, and AT; the final version of the manuscript is approved by all the authors.

## References

[1] Team EE (2020). Note from the editors: World Health Organization declares novel coronavirus (2019-nCoV) sixth public health emergency of international concern. Eurosurveillance..

[2] World Health Organization Iran (Islamic Republic of): WHO Coronavirus Disease (COVID-19) Dashboard With Vaccination Data 2021, December 20.

[3] Naderpour Z, Saeedi M (2020). A primer on covid-19 for clinicians: clinical manifestation and natural course. Frontiers in Emergency Medicine..

[4] Saberian P, Seyed-Hosseini-Davarani S-H, Ramezani M, Mirbaha S, Zangi M, Aarabi S (2022). Concomitant COVID-19 and acute ischemic stroke in patients transferred by emergency medical service during first wave of pandemic in Tehran, Iran; a cross-sectional study. Frontiers in Emergency Medicine.

[5] Tsivgoulis G, Palaiodimou L, Katsanos AH, Caso V, Köhrmann M, Molina C (2020). Neurological manifestations and implications of COVID-19 pandemic. Therapeutic advances in neurological disorders..

[6] Ashrafi F, Ommi D, Zali A, Khani S, Soheili A, Arab-Ahmadi M (2021). Neurological Manifestations and their Correlated Factors in COVID-19 Patients; a Cross-Sectional Study. Archives of academic emergency medicine..

[7] Vakili K, Fathi M, Hajiesmaeili M, Salari M, Saluja D, Tafakhori A ( 2021). Neurological Symptoms, Comorbidities, and Complications of COVID-19: A Literature Review and Meta-Analysis of Observational Studies. European neurology.

[8] Arbabi M, Dezhdar Z, Amini B, Dehnavi AZ, Ghasemi M (2022). Depression and anxiety increase the odds of developing delirium in ICU patients; a prospective observational study. Cognitive Neuropsychiatry..

[9] Shao SC, Lai CC, Chen YH, Chen YC, Hung MJ, Liao SC (2021). Prevalence, incidence and mortality of delirium in patients with COVID-19: a systematic review and meta-analysis. Age and ageing..

[10] Katal S, Gholamrezanezhad A (2021). Neuroimaging findings in COVID-19: A narrative review. Neuroscience letters..

[11] Ladopoulos T, Zand R, Shahjouei S, Chang JJ, Motte J, Charles James J (2021). COVID‐19: Neuroimaging Features of a Pandemic. Journal of Neuroimaging..

[12] Ashrafi F, Zali A, Ommi D, Salari M, Fatemi A, Arab-Ahmadi M (2020). COVID-19-related strokes in adults below 55 years of age: a case series. Neurological Sciences..

[13] Zirpe KG, Dixit S, Kulkarni AP, Sapra H, Kakkar G, Gupta R (2020). Pathophysiological mechanisms and neurological manifestations in COVID-19. Indian Journal of Critical Care Medicine: Peer-reviewed, Official Publication of Indian Society of Critical Care Medicine..

[14] Mao X-Y, Jin W-L (2020). The COVID-19 pandemic: consideration for brain infection. Neuroscience..

[15] Chou SH-Y, Beghi E, Helbok R, Moro E, Sampson J, Altamirano V (2021). Global Incidence of Neurological Manifestations Among Patients Hospitalized With COVID-19—A Report for the GCS-NeuroCOVID Consortium and the ENERGY Consortium. JAMA network open..

[16] Collantes MEV, Espiritu AI, Sy MCC, Anlacan VMM, Jamora RDG (2021). Neurological manifestations in COVID-19 infection: a systematic review and meta-analysis. Canadian Journal of Neurological Sciences..

[17] Metlay JP, Waterer GW, Long AC, Anzueto A, Brozek J, Crothers K (2019). Diagnosis and treatment of adults with community-acquired pneumonia An official clinical practice guideline of the American Thoracic Society and Infectious Diseases Society of America. American journal of respiratory and critical care medicine..

[18] Saeed GA, Gaba W, Shah A, Al Helali AA, Raidullah E, Al Ali AB (2021). Correlation between Chest CT Severity Scores and the Clinical Parameters of Adult Patients with COVID-19 Pneumonia. Radiology research and practice..

[19] Amanat M, Rezaei N, Roozbeh M, Shojaei M, Tafakhori A, Zoghi A (2021). Neurological manifestations as the predictors of severity and mortality in hospitalized individuals with COVID-19: a multicenter prospective clinical study. BMC neurology..

[20] Chen X, Laurent S, Onur OA, Kleineberg NN, Fink GR, Schweitzer F (2021). A systematic review of neurological symptoms and complications of COVID-19. Journal of neurology..

[21] Espiritu AI, Sy MCC, Anlacan VMM, Jamora RDG (2021). COVID-19 outcomes of 10,881 patients: retrospective study of neurological symptoms and associated manifestations (Philippine CORONA Study). Journal of Neural Transmission..

[22] García‐Azorín D, Trigo J, Martínez‐Pías E, Hernández‐Pérez I, Valle‐Peñacoba G, Talavera B (2021). Neurological symptoms in Covid‐19 patients in the emergency department. Brain and Behavior..

[23] Gong X, Kang S, Guo X, Li Y, Gao H, Yuan Y (2021). Associated risk factors with disease severity and antiviral drug therapy in patients with COVID-19. BMC Infectious Diseases..

[24] Zhao C, Bai Y, Wang C, Zhong Y, Lu N, Tian L (2021). Risk factors related to the severity of COVID-19 in Wuhan. International journal of medical sciences..

[25] Dehghanbanadaki H, Aazami H, Shabani M, Amighi D, Seif F, Zare A (2022). A systematic review and meta-analysis on the association between lymphocyte subsets and the severity of COVID-19. immunopathologia persa.

[26] Mao L, Jin H, Wang M, Hu Y, Chen S, He Q (2020). Neurologic manifestations of hospitalized patients with coronavirus disease 2019 in Wuhan, China. JAMA neurology..

[27] Moonis G, Filippi CG, Kirsch CF, Mohan S, Stein EG, Hirsch JA (2021). The Spectrum of neuroimaging findings on CT and MRI in adults with COVID-19. American Journal of Roentgenology..

[28] Chougar L, Shor N, Weiss N, Galanaud D, Leclercq D, Mathon B (2020). Retrospective observational study of brain MRI findings in patients with acute SARS-CoV-2 infection and neurologic manifestations. Radiology..

[29] Lin E, Lantos J, Strauss S, Phillips C, Campion T, Navi B (2020). Brain imaging of patients with COVID-19: findings at an academic institution during the height of the outbreak in New York City. American Journal of Neuroradiology..

[30] Sawlani V, Scotton S, Nader K, Jen J, Patel M, Gokani K (2021). COVID-19-related intracranial imaging findings: a large single-centre experience. Clinical radiology..

[31] Alonazi B, Farghaly AM, Mostafa MA, Al-Watban JA, Zindani SA, Altaimi F (2021). Brain MRI in SARS-CoV-2 pneumonia patients with newly developed neurological manifestations suggestive of brain involvement. Scientific Reports..

[32] Mahammedi A, Ramos A, Bargalló N, Gaskill M, Kapur S, Saba L (2021). Brain and Lung Imaging Correlation in Patients with COVID-19: Could the Severity of Lung Disease Reflect the Prevalence of Acute Abnormalities on Neuroimaging? A Global Multicenter Observational Study. American Journal of Neuroradiology..

[33] Bhatia R, Pedapati R, Komakula S, Srivastava MP, Vishnubhatla S, Khurana D (2020). Stroke in coronavirus disease 2019: a systematic review. Journal of stroke..

[34] Mahammedi A, Saba L, Vagal A, Leali M, Rossi A, Gaskill M (2020). Imaging of neurologic disease in hospitalized patients with COVID-19: an Italian multicenter retrospective observational study. Radiology..

[35] Zochodne DW (2004). SARS, SIRS, and neurological disease. Archives of neurology..

[36] Rostami M, Mansouritorghabeh H (2020). D-dimer level in COVID-19 infection: a systematic review. Expert review of hematology..

[37] MOC C (2020). Coagulopathy in COVID-19: Manifestations and management. Cleveland Clinic journal of medicine..

[38] Chan JL, Gregory KD, Smithson SS, Naqvi M, Mamelak AN (2020). Pituitary apoplexy associated with acute COVID-19 infection and pregnancy. Pituitary..

